# Transparent, Antibiofouling Window Obtained with Surface Nanostructuring

**DOI:** 10.1021/acsomega.4c03030

**Published:** 2024-09-12

**Authors:** Wiktoria K. Szapoczka, Viljar H. Larsen, Hanna Böpple, Dorinde M. M. Kleinegris, Zhaolu Diao, Tore Skodvin, Joachim P. Spatz, Bodil Holst, Peter J. Thomas

**Affiliations:** † University of Bergen, Department of Physics and Technology, Bergen 5007, Norway; ‡ NORCE Norwegian Research Centre AS, Bergen 5008, Norway; § University of Bergen, Department of Biological Sciences, Bergen 5006, Norway; ∥ Department of Cellular Biophysics, Max Planck Institute for Medical Research, Heidelberg D-69120, Germany; ⊥ University of Bergen, Department of Chemistry, Bergen 5007, Norway

## Abstract

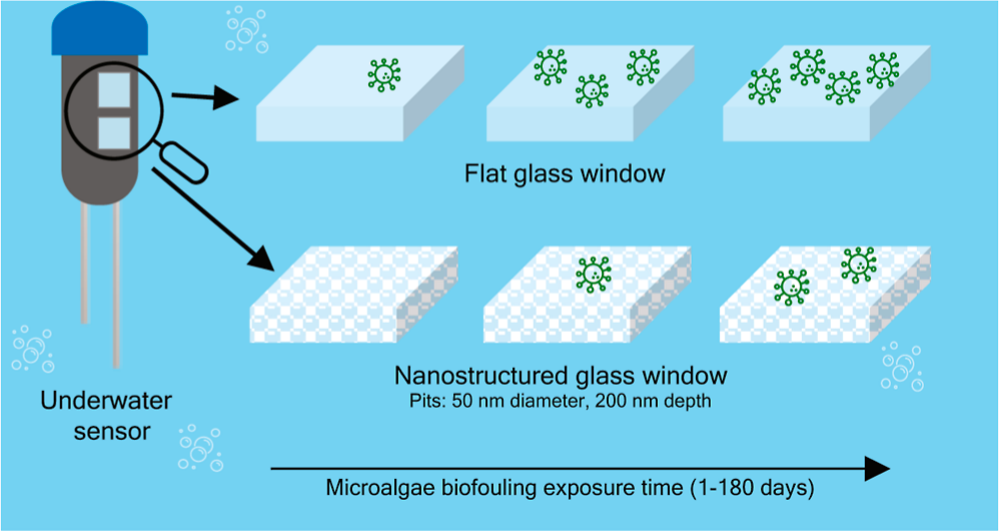

Biofouling is one of the key factors which limits the long-term performance of seawater sensors. Common measures to hinder biofouling include toxic paints, mechanical cleaning and UV radiation. All of these measures have various limitations. A very attractive solution would be to prevent biofilm formation by changing the surface structure of the sensor. This idea has been implemented successfully in various settings, but little work has been done on structuring optically transparent materials, which are often needed in sensor applications. In order to achieve good antibiofouling properties and efficient optical transparency, the structuring must be on the nanoscale. Here, we investigate a transparent, antibiofouling surface obtained by patterning a semihexagonal nanohole structure on borosilicate glass. The nanoholes are approximately 50 nm in diameter and 200 nm deep, and the interparticle distance is 135 nm, allowing the structure to be optically transparent. The antibiofouling properties of the surface were tested by exposing the substrates to the microalgae *Phaeodactylum tricornutum* for four different time intervals. This species was chosen because it is common in the Norwegian coastal waters. The tests were compared with unstructured borosilicate glass substrates. The experiments show that the nanostructured surface exhibits excellent antibiofouling properties. We attribute this effect to the relative size between the structure and the biofouling microorganism. Specifically, the small dimensions of the nanoholes, compared to the biofouling microorganism, make it more difficult for the microalgae to attach. However, lubrication of the substrates with FC-70 perfluorocarbon resulted in contamination at a rate comparable to the reference substrate, possibly due to the chemical attractiveness of the alkane chains in FC-70 for the microalgae.

## Introduction

Seawater sensors are essential for the sustainable exploitation of ocean resources.^1^ Such sensors are often deployed in remote and challenging environments, and end users demand characteristics such as long battery life, high long-term stability, and low maintenance needs.^2^ For marine sensors, biofouling is a major limiting factor for long-term sensor performance.^2−4^

Biofouling is a complex process, defined as unwanted adhesion and buildup of micro- and macroorganisms, plants and animals on submerged surfaces.^5^ It starts within seconds of submersion of a surface in an aquatic environment with the onset of a so-called biofilm formation.^6^ The development process of a biofilm can be divided into five stages. In the initial attachment stage, microorganisms attach to the surface. This can happen through various mechanisms such as physical contact, chemical attraction or biological attraction.^7^ Physical contact happens when microorganisms encounter a surface and settle on top of it, for example, by settling between uneven surfaces. Chemical attraction occurs when microorganisms are attracted by specific chemicals present on the surface, such as alkane chains.^8,9^ Lastly, biological attraction happens as microorganisms are drawn to other microorganisms previously settled on the surface. Once attached, the microorganisms grow, reproduce and produce an extracellular polymeric substance (EPS), further improving the attachment. This provides increased protection against external factors, including penetration of biocides, pH changes and salinity changes.^10^ Such a biofilm poses a good settlement area for macroorganisms, plants and animals, thus continuing the overall biofouling process.

Various antibiofouling solutions have been developed over the years. Traditional solutions, such as toxic paints and transparent coatings, are effective but raise ecological concerns because these paints typically contain nonselective biocides such as copper and tributyltin (TBT).^3,11,12^ Due to the toxicity of biocide-based paints, many countries have implemented strict regulations and bans concerning their use.^3,13,14^ Additionally, the biofouling organisms can become resistant to chemical coatings.^15^ Mechanical cleaning methods are commonly used, including wipers, scrapers, and brushes.^16,17^ These methods are effective but are highly energy-consuming, are a common point of failure, and increase wear on the sensor surface. Mechanical cleaning methods are primarily used on bigger sensors.^3^ More recently, low-cost ultraviolet (UV) irradiation cleaning technologies have been introduced,^3,16,18,19^ despite their effectiveness in reducing biofouling, their energy-intensive nature has posed challenges. Furthermore, irradiation with UV light can damage some sensor components, such as polymers and indicators.^3^

In the search for alternatives, a transition to biofouling prevention at the nanoscale, where the initial attachment of fouling microorganisms occurs, is an attractive option. Nanostructured surfaces offer the potential for targeted fouling prevention without the ecological, energy-consuming, and harmful drawbacks of other solutions.^3,20,21^ Using nanostructured surfaces helps reduce the speed at which microorganisms adhere to the surface, consequently extending the time before costly manual maintenance or sensor replacement is required.

Drawing inspiration from nanostructures found in nature, several antibiofouling solutions based on surface structuring have been proposed.^20,22,23^ The topography of a surface dictates its roughness and wettability, two properties that have been found to affect the production of EPS either by inhibiting or promoting it.^16,20,22^ Already in 2006, over 160 antifouling solutions derived from nature were reported, according to Chambers et al.^23^ Probably the most recognized solution is based on the lotus effect, a water-repelling and self-cleaning effect due to the hierarchical micro and nanostructure of the leaves.^24^ Sharkskin was another early topographic model investigated for antibiofouling properties.^22,25^ The Sharklet AF solution is widely used in industrial applications.^11,22,26,27^ However, these structures are on the microscale and, hence, not suitable for optically transparent surfaces. Control of biofouling adhesion on optical surfaces is challenging since antibiofouling solutions often tend to interfere with optical transparency.^28^ Thus, it is essential to not only understand the antibiofouling properties of a solution but also its optical properties. Some transparent and antibiofouling nanostructures have been proposed, including various slippery lubricant-infused porous surfaces (SLIPS).^29^ Despite good antibiofouling properties and optical transparency, such methods require frequent replenishment of lubricants. Wang et al. (2020) have manufactured a film with nanowires that shows good antibiofouling properties and good transmittance.^30^ Similarly, Han et al. (2018), Akhtar et al. (2018) and Vellwock et al. (2022) have designed and tested nanostructured transparent substrates with good antibacterial and oleophobic properties. Transparent coatings with nanoencapsulation of biocides have also been proposed, and while effective, they can still raise ecological concerns.^31^ As summarized, comparatively little work has been done on making optically transparent antibiofouling substrates.

In the previous work of Diao et al. (2017), the nanostructure found on the eyes of moths inspired the development of a transparent structure on borosilicate glass. The synthesis of nanoholes on both sides of borosilicate glass resulted in greater surface durability. This increase is also the result of the nanostructure being inverted, as opposed to nanopillars with low mechanical stability.^32−34^ The presence of nanoholes also increased optical transmittance from 89% for ordinary glass to 98.5% due to the refractive index gradient created between the air and the surface.^32^ The transmittance and reflectance of the nanostructured substrate were measured using a Cary 5000 Ultraviolet–visible-near-infrared spectrometer, covering a wide range of wavelengths from 175 to 3300 nm. Additionally, fluorinated and lubricated nanostructured substrates were tested, and it was found that there was almost no difference in transmittance observed.^32,35^ This made the surface attractive for use in a study by Zhang et al. (2021) on medical applications. The adhesion of red blood cells and *E. coli* bacteria was investigated using three variations of the substrate: nanostructured borosilicate glass, fluorinated nanostructured borosilicate glass and lubricated nanostructured borosilicate glass. Red blood cells were directly added onto the substrate surface and incubated for 4 h, while *E. coli* bacteria have been suspended in a Lysogeny broth growth medium and inoculated with the substrate for 24 h and three months. All three variations of the substrate show lower adhesion of red blood cells and *E. coli* bacteria compared to the reference substrate (unstructured borosilicate). The lubricated substrates showed the best anticontamination properties.^35^ This was ascribed to the lubricant being infused on the surface and trapped in the nanoholes, lowering the adhesion force between the surface and the contaminant. The lubricant used was FC-70 perfluorocarbon, chosen due to its stability, solvophobicity and biocompatibility.^35,36^ Also, the unlubricated substrates showed good anticontamination properties.

**Figure 1:**
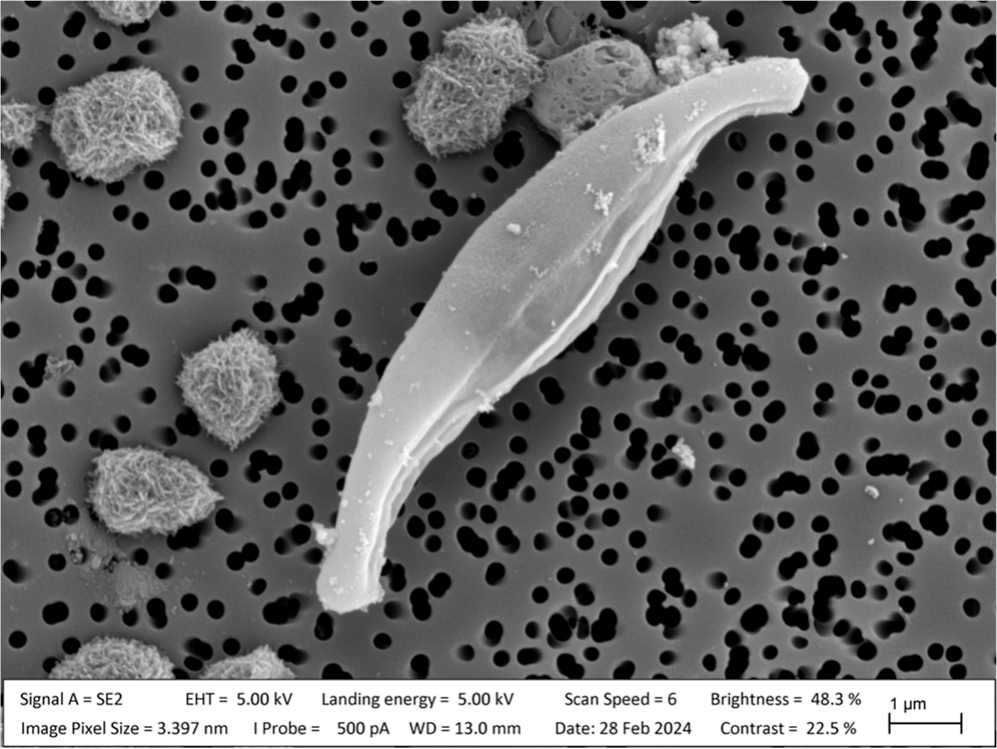
Scanning electron microscopy image of diatom microalgae *Phaeodactylum tricornutum* from B58 strain in the fusiform. Cultured from NORCE, Bergen, Norway.

The results from Diao et al. (2017) and Zhang et al. (2021) inspired the study presented in this paper, where we test the exact same type of surfaces for their antibiofouling properties when being exposed to biofouling by microalga *Phaeodactylum tricornutum* (Figure 1).

*P. tricornutum* is a diatom with a length between 18 and 26 μm and a width between 2 and 3 μm, commonly found in diverse marine environments, including pelagic and benthic habitats. Its cell wall is mainly organic, and most strains can show three interchangeable morphotypes: ovoid, fusiform and triradiate. *P. tricornutum* was chosen as the biofouling microorganism for two reasons: 1. its common presence in the heavily monitored Norwegian coastal waters, which makes it particularly industrially relevant, and 2. the use of this microalga in other biofouling studies on nontransparent substrates.^9,37,38^ By focusing on one commonly found biofouling organism, the analysis of the antibiofouling properties of the surface is simplified. It has been reported that different *P. tricornutum* strains show different adhesion characteristics, with not all strains forming strong biofilms, likely based on the various types of EPS produced by the cells.^39^ Moreover, it is the ovoid forms of *P. tricornutum,* in particular, that are adhesive.^40^ Although this strain (B58) is not used in those studies, *P. tricornutum* B58 is capable of showing all three morphotypes, and it has been shown that this strain forms strong biofilms both on submerged and nonsubmerged carrier material (Böpple et al. submitted for publication). Avelelas et al. (2017) and Figueiredo et al. (2019) have previously utilized this microalga in biofouling tests of biocides embedded in nanostructured surfaces. Yue et al. (2023) have used *P. tricornutum* to test the antibiofouling properties of hierarchical micro- and nanostructures on titanium alloy. The surface consisted of micropores (1.5 ± 0.3 μm) with and without additional nanostructuring. Here, as well, different variations of the substrates were tested: fluorinated and lubricated, both with and without the nanostructuring present. The lubricant used was perfluoropolyether (PFPE). The results showed the antibiofouling properties of the substrates, with the substrate having nanoprickles and an infused lubricant obtaining the best antibiofouling results.

## Results and Discussion

### Characterization of the Substrates

**Figure 2:**
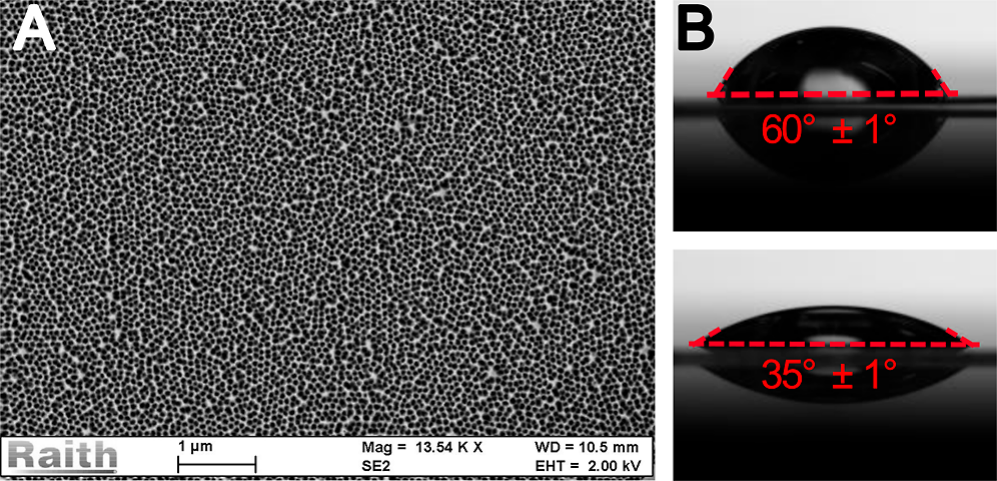
(A) Scanning electron microscopy image of the surface morphology of a nanostructured glass. (B) Optical images of a 10 μL super distilled water droplet on a reference glass (top) and nanostructured glass (bottom) and the corresponding optical contact angles.

The nanostructured substrates were synthesized following the method described by Diao et al. (2017), which can be found in the experimental section. In total, eight individual substrates are tested. Three reference substrates, three nanostructured substrates, one fluorinated substrate and one lubricated substrate. The nanostructured pattern on the surface was characterized by a scanning electron microscope (SEM). Figure 2A shows the substrate surface. The nanoholes are evenly distributed, and no obvious defects to the surface are visible. The average interparticle distance is 100 ± 20 nm. This result compares well with the previously reported distance of 135 ± 32 nm.^35^ The nanoholes are 50 nm in diameter and 200 nm deep, as previously reported by Zhang et al. (2021). Optical contact angle (OCA) measurements were conducted to investigate the wettability change of the surface after structuring. Figure 2B shows the OCA of 60 ± 1° for the reference substrate (top) and 35 ± 1° for the nanostructured substrate (bottom). These measurements agree with the previously reported values of 60 ± 1° and 33 ± 1°, respectively.^35^ The OCA results show that the nanostructured substrate is more hydrophilic than the reference substrate, a necessary change in order to obtain the underwater oleophobicity and improve the antibiofouling properties of the surface.^1^ Surface energy (chemical composition) and surface roughness (structuring) are the two factors that determine the wettability of a solid surface.^9^ Here, the decrease of the OCA can be attributed to the capillary effect caused by the presence of the nanoholes at the surface, consequently increasing the surface roughness.^35,41,42^ Excessive surface roughness can lead to incomplete air entrapment, and liquid may penetrate the nanostructure, leading to a lower contact angle. A table with all the OCA data can be found in the Supporting Information (Table S1).

### Evaluation of Antibiofouling

**Figure 3:**
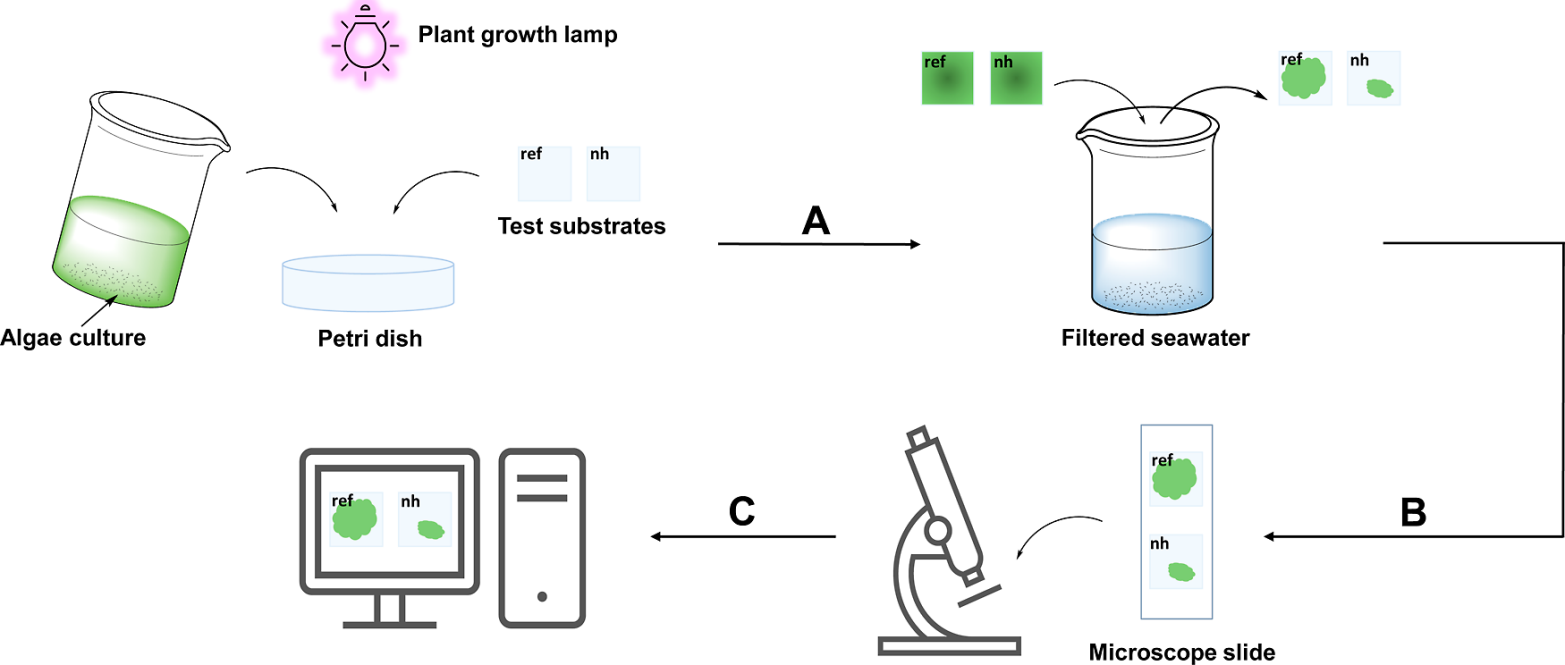
Schematic of the antibiofouling experiment. The substrates (ref: reference substrate; nh: nanostructured substrate) are placed in a Petri dish filled with the microalgae stock and stored under UV growing lights for either 1 day, 7 days, 21 days or 180 days. (A) After a specific amount of time, the substrates are gently washed in freshly filtered seawater for 15 s by stirring so that the loose microalgae are removed, and only the sticking microalgae are left on the surface to evaluate further. (B) The substrates are placed on a microscope slide for optical analysis. (C) The images obtained are further used in statistical analysis using ImageJ software.

**Figure 4:**
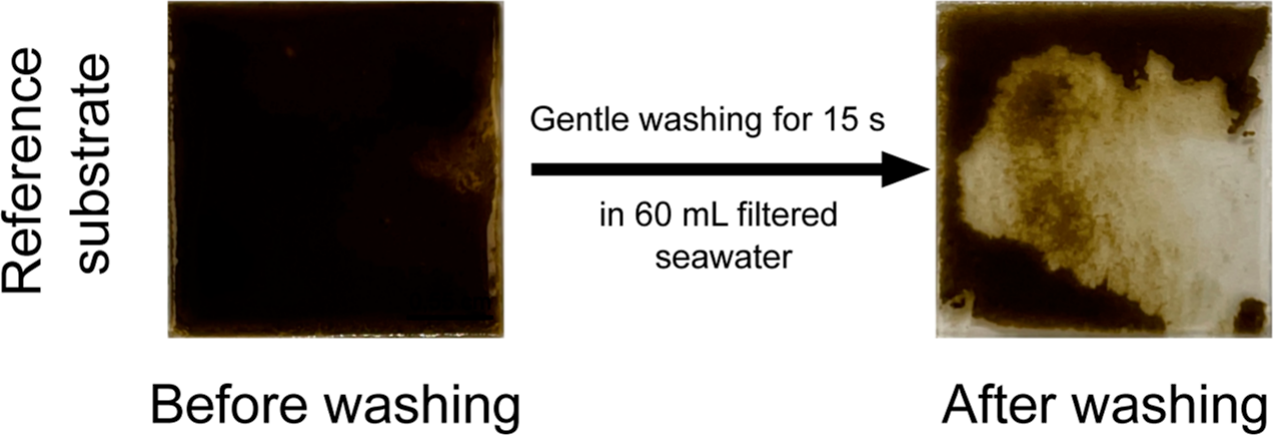
Reference substrate before (left) and after (right) washing for 15 s in 60 mL of freshly filtered seawater after submersion in the algal stock for 21 days.

The antibiofouling tests were performed as described in the experimental section and shown in Figure 3. The substrates were placed in a Petri dish and submerged in microalgae stock solution for a specific amount of time. The substrates were then gently washed with distilled water and analyzed. Figure 4 shows images of a reference substrate that has been submerged for 21 days before and after washing.

**Figure 5:**
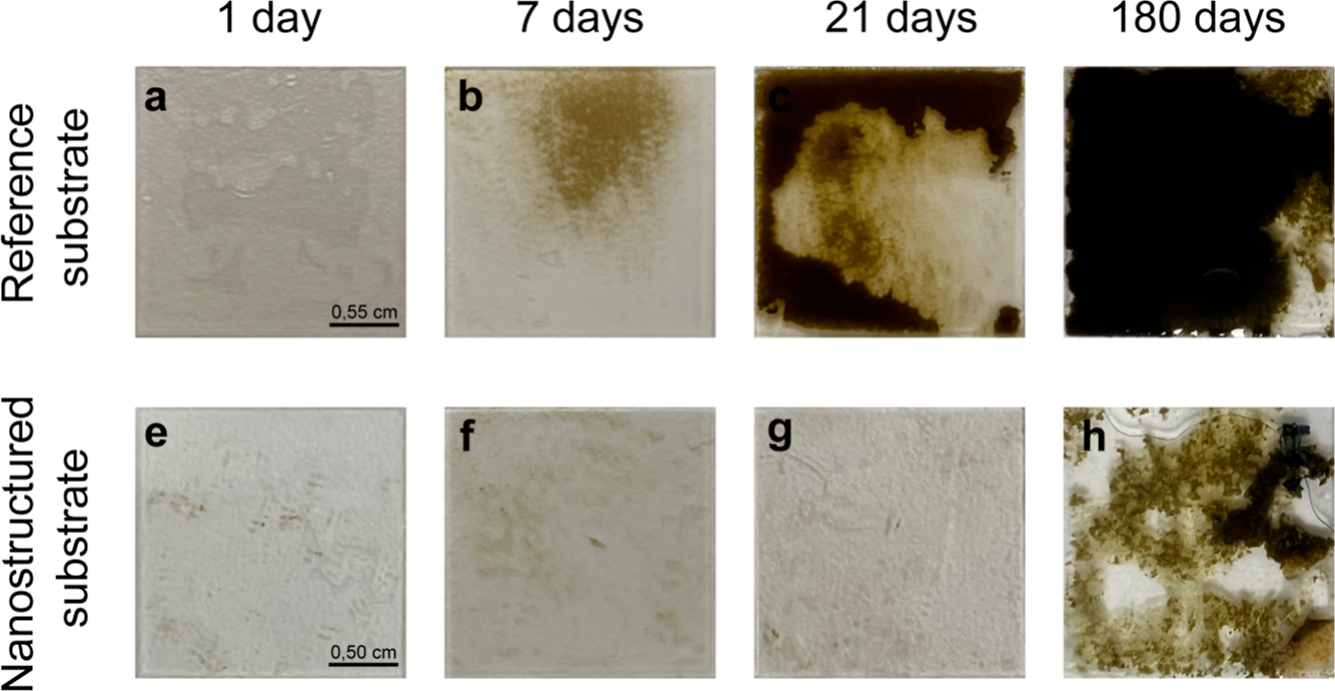
Adhesion of microalgae *Phaeodactylum tricornutum* to the test substrates after the washing step. Top row: reference substrate. Bottom row: nanostructured substrate. Each column shows the corresponding substrates after a certain amount of time: (a,e) after 1 day, (b,f) after 7 days, (c,g) after 21 days and (d,h) after 180 days.

Figure 5 shows the optical images obtained from the biofouling exposure tests of a reference substrate and a nanostructured substrate after 1-180 days of exposure to contamination and subsequent washing step. The top row, panels a–d, shows the reference substrates. The bottom row, panels e–f, shows the nanostructured substrates. The amount of days for each sampling increases from left to right (1-180 days). The reference substrate is strongly contaminated compared to the nanostructured substrate. After 7 days, there is already a visible difference in the algal growth between the two substrates.

**Figure 6:**
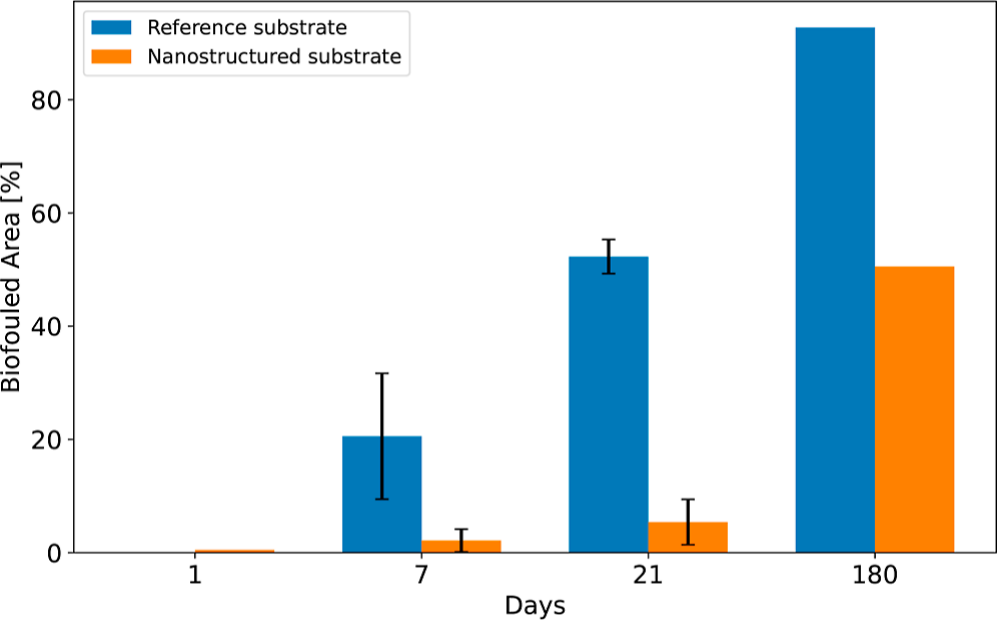
Percentage biofouled area for the different time intervals (1-180 days). Blue: reference substrate. Orange: nanostructured substrate. The error bars represent the mean ± standard error of three independent experiments’ mean (s.e.). No error bar is depicted for Day 1, as the s.e. is 0%. For 180 days, only one nanostructured and one reference substrate were tested. Detailed data can be found in the Supporting Information.

The obtained images were further analyzed using ImageJ, and the biofouling adhesion percentage surface coverage was quantified. A statistical analysis of the biofouling growth over time is shown in Figure 6. The biofouling tests were performed on three identical reference substrates and three identical nanostructured substrates for shorter time intervals with good repeatability. The test for 180 days was only performed once, and thus, the results cannot be presented with error bars. As expected, the longer the time interval, the more biofouling is present on the substrates. However, the reference substrate exhibits a much stronger increase in biofouling colonisation. 20% ± 10% after 7 days, 52% ± 3% after 21 days, and a substantial 93% after 180 days. In contrast, the nanostructured substrate shows consistent resistance to biofouling adhesion at 2% ± 1% after 7 days, 5% ± 4% after 21 days and 51% after 180 days. Data used to calculate these values can be found in the Supporting Information (Tables S3 and S4). In addition to the traditional optical images, the biofouling was also investigated with a fluorescence microscope. The obtained images can be found in the Supporting Information (Figure S1). The captured images focus on a smaller substrate area compared to the images in Figure 5. From the visual data, it is possible to see that more microalgae have attached to the reference substrate than to the nanostructured substrate after 7 days. After 21 days, it is possible to see that the fluorescence of the biofouling on the reference substrate is less intense. Since the substrates are lit from underneath, the loss in intensity shows that the microalgae are more densely packed after 21 days due to an increase in biofouling, thus letting less excitation light through. Not much change in the biofouling growth and density can be observed for the nanostructured substrate.

Based on these results, it can be concluded that the nanostructured substrate exhibits long-term antibiofouling properties against *P. tricornutum*. Moreover, the structuring increases the surface roughness, simultaneously making the surface more hydrophilic. It has previously been reported that the topography of a surface affects the attachment of biofouling agents based on the size proportion of the structuring and the biofouling microorganisms.^20,21,30,43^ In general, as long as the pattern on the substrate surface has dimensions smaller than the size of the biofouling microorganisms, the surface area available for the microorganisms to adhere to is reduced.^20,30^ This results in a decreased probability of interaction and attachment to the surface, further resulting in reduced biofilm and EPS formation. For microalgae, it is easier to attach to a flat surface, such as borosilicate glass, than to a uniformly nanopatterned substrate. The presence of holes reduces the fraction of the surface area available for settlement of the microalgae, as there is not enough space coverage for the microalgae to secure themselves onto. The microalgae are then rather slightly sitting on top of the nanoholes instead of attaching themselves to the surface. Wang et al. (2020) have observed a similar relationship between the size of the nanostructure and the biofouling organism. The origin of such a size effect can be attributed to the need for a uniform surface nature in order to attach and produce EPS properly. This phenomenon explains well the antibiofouling properties of the nanostructured substrates, as the dimensions of the nanoholes are much smaller (diameter: 50 nm, depth: 200 nm, interparticle distance: 135 nm) compared to the size of the microalgae (length: 18–26 μm, width: 2–3 μm).

After some time, biofouling can also be observed on the nanostructured substrates (Figure 6). The reason for that can be a possible buildup of EPS, as more and more microalgae attach. Fluorination of the nanostructured substrate has previously shown good results in terms of antifouling.^35^ Fluorination can be used as a chemical treatment of the surface that will lower the surface energy,^34,44^ which again affects the wettability of the surface. One fluorinated substrate was tested against biofouling, and after 21 days, only 1% of the substrate area was contaminated, which is lower than the 5% obtained for the nanostructured substrate (Figures S2 and 5g). The contact angle of the fluorinated substrate was previously measured to be 132°.^35^ This shows that the surface went from being hydrophilic to hydrophobic. This change can be attributed to the mix of increased surface roughness and surface energy reduction,^34,44^ which, when combined, can enhance air trapping at the surface, permitting the establishment of the Cassie–Baxter state. In this state, air pockets between the solid and the liquid are present at the surface, acting as a protective antibiofouling layer.^9,30,34,44^ This isolation barrier of air makes it impossible for the biofouling microorganisms to attach to the surface.

A theoretical calculation of the Cassie–Baxter angle for the fluorinated surface has been performed. The value we calculated (150°) agrees with the value obtained by Zhang et al. (2021) (132°). In our measurements, we obtained a CA that is slightly smaller, which can be attached to the quality of the fluorination. The calculation of the theoretical Cassie–Baxter angle can be found in the Supporting Information. Fluorination seems to allow the establishment of the Cassie–Baxter state at the surface, but it will not eliminate the dissolving of air in water.^9^ The reason is that after long-term immersion in water, the trapped air will start to dissolve, and the surface will lose its protective barrier.^9,45^ Additionally, it is energetically more profitable for water to fill the nanoholes, causing the metastable Cassie–Baxter state to return to the Wenzel state observed for the solely nanostructured substrate.^34^

Lubricating the fluorinated nanostructured substrates previously yielded the best results against the adhesion of red blood cells and *E. coli* compared with the solely nanostructured and fluorinated substrates.^35^ The same tendency was found by Yue et al. (2023) for structured titanium alloys exposed to *P. tricornutum*. The use of lubricant lowers the surface energy further, stabilizing the Cassie–Baxter state yet more.^9,44^ In this study, one lubricated nanostructured substrate was tested against biofouling. 37% and 45% of the surface were contaminated after 7 and 21 days, respectively (Figure S1). This contamination percentage is similar to the one present at the reference substrate (Figure 5b,c). The lubricant used was FC-70 perfluorocarbon, the same as for the tests performed on the nanostructured substrate against red blood cells and *E. coli*.^35^ Perfluorocarbon FC-70 is a tertiary amine made of fluorinated alkane chains, and while the presence of the negatively charged fluorine atoms can repel microorganisms by repulsive Coloumb forces,^9,46^ the alkane chains may be chemically attractive for biofouling microorganisms and thus accelerate the colonisation of the surface.^8,9^ Furthermore, biofilm formation at a liquid–liquid interface differs from the previously described biofilm formation at a solid–liquid interface, affecting, i.e. the growth and the structure of the biofilm.^8^ The hydrophobicity of biofouling microorganisms plays a crucial role in adsorption to oil–water interfaces, such as the one obtained when using an oil-based lubricant on the substrate surface.^8,47^

Both the ovoid and fusiform morphotypes of *P. tricornutum* secrete EPS in the form of carbohydrates, proteins and sulfates.^48^ Growth conditions can influence the EPS composition; for example, stress conditions can lead to an enrichment of highly branched/substituted and terminal rhamnose, xylose, and fucose, as well as *O*-methylated sugars, uronic acids, and sulfate in the cell walls of fusiform *P. tricornutum*.^49^ Abdullahi et al. (2006) suggested that this enrichment can increase the hydrophobicity and the cross-linking in the cell wall to protect the cells from stressful environmental conditions. Willis et al. (2013) suggest that the linkage of the monosaccharides is especially important for the adhesive function of the mucilage and not necessarily the monosaccharide composition.

Stanley and Callow (2007) showed that some *P. tricornutum* strains had greater adhesion strength on a hydrophobic surface (Silastic T2 silicone elastomer) than on hydrophilic acid-washed glass, as is seen here, where *P. tricornutum* experienced an affinity to perfluorocarbon FC-70.

Correspondingly, hydrophilic red blood cells were not attracted to perfluorocarbon FC-70 when the anticontamination properties of the nanostructured substrate were investigated by Zhang et al. (2021), explaining the excellent results. Nevertheless, biofilm formation at oil–water interfaces is a complex process that is not solely determined by the hydrophobic effect.^8^ The growth and strength of biofilms at liquid interfaces can be influenced by various additional factors, including the presence of secreted biosurfactants (surface-active molecules produced by microorganisms) and metabolic factors. Depending on the bacterial strain, *E. coli* bacteria is hydrophobic, allowing the bacteria to adhere to the liquid interface.^47^ Nevertheless, the formation of biofilm is also strongly dependent on the ability of the bacteria to thrive on and metabolize the organic phase. *E. coli* has been previously shown to hardly adsorb to an oil–water interface, forming a scattered structure that only partially covers the interface, and the viscoelastic properties of this biofilm were mainly attributed to the presence of proteins.^47^ This supports the anticontamination properties of the nanostructured substrate against *E. coli* obtained by Zhang et al. (2021). Marine biofouling occurs in a different environment compared to blood and bacteria contamination in the body, which was previously tested by Zhang et al. (2021). Microalgae, bacteria, and blood cells are three different types of organisms, consequently causing biofouling in diverse ways. This is evident from how the microalgae reacted to the lubricated substrates compared to bacteria and red blood cells, which shows that there is no uniform solution to the problem.

## Conclusion and Future Perspectives

The findings presented in this paper demonstrate the effective antibiofouling properties of moth-eye-inspired, transparent nanostructured substrates. The material’s resilience against algal colonisation is particularly noteworthy, even during extended exposure. This antibiofouling property can be ascribed to the topography of the surface. The presence of nanoholes increases the hydrophilicity of the surface and enhances the surface roughness. The dimensions of the nanoholes, being smaller than the biofouling microorganism, make it more difficult for the microalgae to attach. By fluorinating the nanostructured surface, the antibiofouling properties improved further. This can be explained by the possible establishment of the advantageous Cassie–Baxter state due to surface energy reduction implemented by the fluor atoms. The presence of the air layer between the solid and liquid acts as a protective antibiofouling barrier. Lubrication with perfluorocarbon FC-70 led to contamination at a rate similar to that of the reference substrate. We attribute this to the alkane chains, which are known to be chemically attractive for biofouling microorganisms. Future endeavors will extend to field testing, providing a real-world assessment of these materials in dynamic contamination scenarios.

## Experimental Section

### Materials

A local isolate of *P. tricornutum* was used (B58), originating from Bergen, Norway (called N58 in previous publications^50^). *P. tricornutum* was grown on filtered seawater enriched with a stock solution, resulting in the following concentrations (in mM): NaNO_3_, 25; KH_2_PO_4_, 1.7; Na_2_EDTA, 0.56; Fe_2_SO_4_·7H_2_O, 0.11; MnCl_2_·2H_2_O, 0.01; ZnSO_4_·7H_2_O, 2.3 × 10^–3^; Co(NO_3_)_4_·6H_2_O, 0.24 × 10^–3^; CuSO_4_·5H_2_O,0.1 × 10^–3^; Na_2_MoO_4_·2H_2_O, 1.1 × 10^–3^. The medium was adapted from de Vree et al.^51^ (2016), and the materials were obtained from Sigma-Aldrich. The microalgae were cultivated in 2 L DURAN borosilicate flasks under continuous stirring (50–100 rpm) and under LED light conditions, light/dark photoperiod of 12 h:12 h at 21 ± 2 °C. Illumination occurred from above, and the lamp used was a white LED (230 V, 14 W, PAR photons, photosynthetic photon flux 14 μ mol/s). The algal culture stock was subcultured every 4 weeks. Seawater was collected from Damsgaardssundet, Bergen, Norway (lat. 60°N, long. 5°E) and filtered through a 0.8/0.2 μm AcroPak 500 filter capsule (Cytiva). Seawater had a salinity of 32-33 ppt. 1H, 1H, 2H, 2H-perfluorodecyltrichlorsilane, perfluorocarbon FC-70 and borosilicate coverslips were obtained from Sigma-Aldrich. All glassware was sterilized and flushed with filtered seawater before use. Ultrapure water (Milli-Q) was used for optical contact angle measurements.

### Synthesis and Characterization of the Substrates

The synthesis of the nanostructured substrates went as described by Diao et al. (2017). First, a hexagonal layer of gold nanoparticles on borosilicate coverslips (22 mm × 22 mm) was generated using Block Copolymer micellar lithography (BCML). The gold nanoparticles were then enlarged by electroless deposition, and a 5 nm chromium layer was sputtered onto the substrate. Next, the gold nanoparticles were removed from the surface using piranha solution (H_2_SO_4_ (98%)/H_2_O_2_ (30%) = 3:1) and a chromium layer with a semihexagonal became visible. This process was followed by reactive ion etching (RIE) leading to the formation of nanoholes on the substrate. To fluorinate the nanostructured substrate, the substrate was put under vacuum together with 1H, 1H, 2H, 2H-perfluorodecyltrichlorsilane vapor for 30 min and placed in an oven at 80 °C for 2 h to stabilize the binding. In order to obtain a lubricated substrate, a fluorinated substrate was dipped in lubricant oil perfluorocarbon FC-70, with the excess oil removed by standing the substrate with tweezers. The presence of nanoholes on an uncoated substrate was imaged in a Raith e-Line EBL (electron beam lithography) system, which has an SEM imaging system based on Zeiss (Gemini) SEM. The wettability of the substrates was analyzed using the sessile drop method by the OCA 20 instrument from Dataphysics. Ultrapure water droplets of 10 μL were used for the characterization, and the measurements were performed at 22 °C. Untreated borosilicate coverslips were used as reference substrates.

### Biofouling Setup and Characterization

The microalgae were examined with a Zeiss Gemini 450 SEM. The biofouling tests were executed under the same conditions as the algal cultivation, except for the stirring not taking place; see the section above and Figure 3. Petri dishes were filled with the algal stock (15 mL). Three nanostructured substrates and three reference substrates were submerged in pairs (one nanostructured, one reference) in a Petri dish filled with the stock and incubated for three different time intervals: 1 day, 7 days, and 21 days. One of the nanostructured substrates and one of the reference substrates were additionally incubated for 180 days. Furthermore, one fluorinated substrate and one lubricated substrate were investigated. In total, eight substrates were investigated in this study. The Petri dishes were half-covered with a glass cover in order to lessen the evaporation of the stock, simultaneously enabling air circulation necessary for the algal growth. Each week, the Petri dishes were replenished with the algal stock to compensate for the evaporation. The six substrates were then taken out and washed by stirring for approximately 15 s in a beaker filled with freshly filtered seawater (60 mL). The washed substrate was then placed on a microscope glass on a white background. The algal adhesion was observed from above by taking optical images, which were further analyzed and quantified in percentage with the freeware ImageJ software developed at the National Institutes of Health, Bethesda, Maryland. All data are presented as the mean ± standard error of three independent experiments’ mean (s.e.) unless otherwise stated.

## Supporting Information Available

Supporting Information OCA measurements; the adapted seawater medium recipe; two tables containing biofouled areas by percentage of reference and nanostructured substrates; fluorescence microscopy images of the reference and nanostructured substartes after 7 and 21 days; images of the fluorinated and lubricated substrates and their corresponding biofouled areas in percentage; Cassie–Baxter equations (PDF). The following files are available free of charge. (PDF)
