# Supplementary material for: Transparent, Antibiofouling Window Obtained with Surface Nanostructuring
Source: ACS Omega. 2024 Sep 12;9(38):39464–71. doi: 10.1021/acsomega.4c03030 (PMC11425857; doi:10.1021/acsomega.4c03030)
Supplement: Supplementary file 1 — ao4c03030_si_001.pdf [file ao4c03030_si_001.pdf]

Supporting Information

A transparent, anti-biofouling window obtained with surface nanostructuring

Wiktoria K. Szapoczka,[*,†] Viljar H. Larsen,[†] Hanna Böpple,[‡] Dorinde M. M. Kleinegris,[¶,‡] Zhaolu Diao,[§] Tore Skodvin,[//] Joachim P. Spatz,[§] Bodil Holst,[†] and Peter J. Thomas[*,‡]

† *University of Bergen, Department of Physics and Technology, Bergen, 5007, Norway*

‡ *NORCE Norwegian Research Centre AS, Bergen, 5008, Norway*

¶*University of Bergen, Department of Biological Sciences, Bergen, 5006, Norway*

§*Department of Cellular Biophysics, Max Planck Institute for Medical Research, Heidelberg, D-69120, Germany*

//*University of Bergen, Department of Chemistry, Bergen, 5007, Norway*

E-mail: wiktoria.szapoczka@uib.no; peth@norceresearch.no

Optical contact angle measurements

Table S1. Optical contact angle values for the reference substrate and nanostructured substrate.

Measurement	Reference substrate [°]	Nanostructured substrate [°]
1	62	34
2	60	35
3	64	30
Average	62	33
Standard deviation	2	3

Seawater medium

The stock solution of trace mineral and nitrate substrate described below was added as a nutrition enhancer for the algal stock culture. The seawater medium was prepared in a sterilised bottle and stored at 4°C.

Table S2. Seawater medium recipe used in the cultivation of biofouling microalgae *Phaeodactylum tricornutum*.

**Trace mineral stock**	**Conc. in medium**	**per liter stock solution**
$Na_2EDTA*H_2O$	282 µM	45 g
$FeSO_4$, 7 H_2O	108 µM	30 g
$MnCl_2$, $2H_2O$ or:	11 µM	1.71 g
$MnCl_2$, $4H_2O$	11 µM	2.09 g
$ZnSO_4$, 7 H_20	2.3 µM	0.66 g
$Co(NO_3)_2$, 6 H_20	0.24 µM	70 mg
$CuSO_4$, 5 H_20	0.1 µM	24 mg
Na_2MoO_4,$2H_20$	1.1 µM	242 mg

**Nitrate-substrate**	**Conc. in medium**	**per liter substrate**
$NaNO_3$	25 mM	106 g
KH_2PO_4	1.7 mM	11.5 g
Na_2EDTA	173 µM	3 g
Trace mineral stock		50 ml
Dissolve in demi water and make a total volume of 1000 ml.		1000 ml
Adjust pH to 7.5-7.6 with NaOH		

	**Substrate per liter medium**
**Medium 5 - nitrate**	20 ml

Reference and nanostructured substrates

Table S3. Biofouled areas by percentage after 1 – 21 days of the three reference substrates together with average and standard deviation.

Days	Reference substrate 1	Reference substrate 2	Reference substrate 3	Average	Standard deviation [± %]
	Biofouled area [%]				
1	0	0	0	0	0
7	0,2	23	39	21	11
21	49	54	54	52	3
180	-	93	-	-	-

Table S4. Biofouled areas by percentage after 1 – 21 days of the three nanostructured substrates together with average and standard deviation.

Days	Nanostructured substrate 1	Nanostructured substrate 2	Nanostructured substrate 3	Average	Standard deviation [± %]
	Biofouled area [%]				
1	0,8	0	0	0	0
7	3	0,2	3	2	1
21	10	1	5	5	4
180	-	51	-	-	-

The substrates were also investigated with a Dino-lite microscope, and the attached algae were excited with a Thorlabs UV LED (M365L2 - 365 nm, 190 mW (Min) Mounted LED, 700 mA). Compared to the optical images shown in the main paper, which show the whole area of the substrate, these images only focus on a small part of the surface.

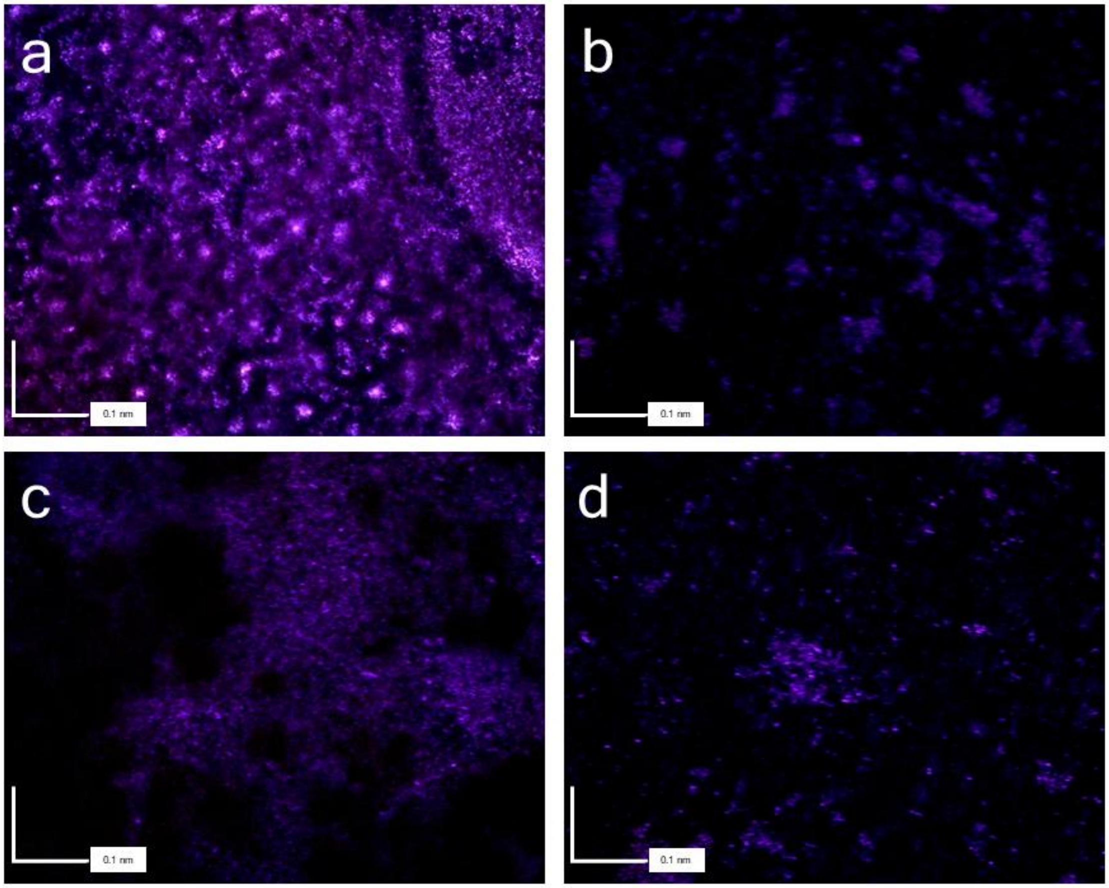

Figure S1. Microscope images of UV-illuminated substrates after exposure to biofouling microalgae *Phaeodactylum tricornutum*. a) reference substrate after 7 days of exposure, b) nanostructured substrate after 7 days of exposure, c) reference substrate after 21 days of exposure and d) nanostructured substrate after 21 days of exposure.

Fluorinated and lubricated substrates

While calculating the biofouled area percentage for the fluorinated substrate (Figure S1, left), the biofouled areas on the surface corners were ignored. This contamination can be attributed to scratches on the surface obtained while handling the sample with metal tweezers, as opposed to soft plastic tweezers used for the nanostructured substrates.

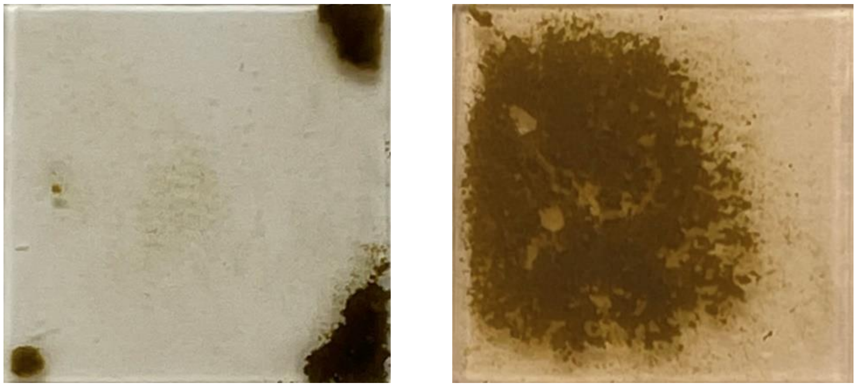

Figure S2. Left: fluorinated substrate and right: lubricated substrate after 21 days of exposure to biofouling microalgae *Phaeodactylum tricornutum*.

Table S5. Biofouled areas by percentage after 1 – 21 days of the two additional substrates. The value marked with * has been obtained by ignoring biofouling gained from scratching the surface corners.

**Days**	**Fluorinated substrate**	**Lubricated substrate**
	**Biofouled area [%]**	
1	0	1
7	15	37
21	1*	45

Cassie-Baxter calculation

The Cassie-Baxter equation (*Eq. S1*) is used to calculate the Cassie-Baxter angle (θ_{CB}). The experimental contact angle of a fluorinated reference substrate is used as the intrinsic Young's contact angle (θ_Y). Here, f_s and f_v are the solid air pocket fractions, respectively.

$$\cos\theta_{CB} = f_s \cos\theta_Y - f_v \qquad \textbf{Eq. S1}$$

To calculate the surface fractions, the surface area of a unit cell must be determined. The following dimensions for the nanohole are used:

Diameter (d): 90 nm

Spacing between the nanoholes (s): 135 ± 32 nm

Intrinsic Young's contact angle (θ_Y): 94°

The area of a nanohole is calculated by *Eq. S2*:

$$A_{nh} = \pi\left(\frac{d}{2}\right)^2 = \pi\left(\frac{90\ nm}{2}\right)^2 \approx 6361{,}73\ nm^2 \qquad \textbf{Eq. S2}$$

Nanoholes are in a hexagonal pattern, so consequently, the unit cell is a hexagon, with each side equal to the spacing between the centers of adjacent holes. The area of a hexagon with side length, *s*, is calculated by *Eq. S3*.

$$A_{hex} = \frac{3\sqrt{3}}{2}s^2 = \frac{3\sqrt{3}}{2}(135\ nm)^2 \approx 47349{,}94\ nm^2 \qquad \textbf{Eq. S3}$$

The solid fraction is given by *Eq. S4*:

$$f_s = \frac{A_{nh}}{A_{hex}} = \frac{6361{,}73\ nm^2}{47349{,}94\ nm^2} \approx 0{,}1344 \qquad \textbf{Eq. S4}$$

The air pocket fraction is given by *Eq. S5*:

$$f_v = 1 - f_s = 1 - 0{,}1344 = 0{,}8656 \qquad \textbf{Eq. S5}$$

Calculating Cassie-Baxter angle:

$$\cos\theta_{CB} = 0{,}1344 \cdot (-0{,}0698) - 0{,}8656 = -0{,}8750$$
$$\theta_{CB} = \cos^{-1}(-0{,}8203) \approx 151°$$

Based on these results, the surface can exhibit a high apparent contact angle of 151º, indicating hydrophobic behaviour. This is an increase from the intrinsic contact angle of 94°, demonstrating the impact of fluorination combined with nanostructuring in the possibility of achieving the Cassie-Baxter state. It is important to note that the theoretical dimensions of nanoholes are used to calculate the fractions.
